# Exploring the Impacts of Sorghum (*Sorghum bicolor* L. *Moench*) Germination on the Flour’s Nutritional, Chemical, Bioactive, and Technological Properties

**DOI:** 10.3390/foods13030491

**Published:** 2024-02-03

**Authors:** Diogo Salvati, Beatriz Helena Paschoalinotto, Filipa Mandim, Isabel C. F. R. Ferreira, Nádia Cristiane Steinmacher, Carla Pereira, Maria Inês Dias

**Affiliations:** 1 Centro de Investigação de Montanha (CIMO), Instituto Politécnico de Bragança, Campus de Santa Apolónia, 5300-253 Bragança, Portugal; diogosalvati@alunos.utfpr.edu.br (D.S.); paschoalinotto@ipb.pt (B.H.P.); filipamandim@ipb.pt (F.M.); iferreira@ipb.pt (I.C.F.R.F.); carlap@ipb.pt (C.P.); 2 Laboratório Associado Para a Sustentabilidade e Tecnologia em Regiões de Montanha (SusTEC), Instituto Politécnico de Bragança, Campus de Santa Apolónia, 5300-253 Bragança, Portugal; 3 Departamento Acadêmico de Alimentos (DAALM), Campus Medianeira, Universidade Tecnológica Federal do Paraná, Medianeira 85884-000, Brazil; nadiac@professores.utfpr.edu.br

**Keywords:** sprouted sorghum flour, technological properties, nutritional characterization, bioactivity

## Abstract

Germination is a natural, simple, and economical process used to improve the quality of nutritional and technological grains. In this study, native and sprouted sorghum flours were characterized regarding their technological properties (particle size distribution, water, and oil absorption capacity, swelling power and solubility, microscopy of starch granules, and pasting and thermal properties). Nutritional and phytochemical characterization profiles, including free sugars, fatty acids, organic acids, tocopherols, and phenolic compounds, were explored through chromatographic methods. The antioxidant, anti-inflammatory, and cytotoxic activities of the respective hydroethanolic extracts were also evaluated. The results showed that the germination process caused significant changes in the flour composition and properties, causing reduced gelatinization temperature and retarded starch retrogradation; an increased content of free sugars and total organic acids; and a decreased content of tocopherols and phenolic compounds. In terms of bioactivity, the sprouted sorghum flour extract showed better lipid-peroxidation-inhibition capacity and none of the extracts revealed hepatotoxicity or nephrotoxicity, which are important results for the validation of the use of the flours for food purposes. Germination is an efficient and alternative method for grain modification that gives improved technological properties without chemical modification or genetic engineering.

## 1. Introduction

Sorghum (*Sorghum bicolor* (L.) Moench) holds the fifth position among the most crucial cereal crops globally, following wheat, rice, corn, and barley [1]. Sorghum is a hardy crop with a high tolerance of water deficit, making it suitable for cultivation in various seasons and regions with arid and semi-arid climates [2]. It is of great importance globally, particularly in African and Asian countries, where it is a primary source of protein and is considered a staple food for millions of people [3,4]. Furthermore, in countries such as the United States, Mexico, Argentina, Brazil, and Australia, it has conventionally been used as animal feed [5].

Grain consists of three biological components: the pericarp, endosperm, and germ. According to genotypes and production types, the distribution of these components varies. On average, the pericarp, endosperm, and germ make up approximately 7%, 84%, and 9% of the grain weight, respectively [6]. The nutritional composition of sorghum grain is directly influenced by climatic, environmental, and genetic factors. Generally, the grain contains 55% to 77% starch, 7% to 19% protein, 1% to 5% fat, 8.5% to 24% fiber, 1% to 2.8% ash, and 9% to 11% moisture [7].

Sorghum grain has been recognized as a promising reservoir of phenolic compounds, underscoring the significance of incorporating it into diets for the prevention and regulation of chronic diseases. This association is directly linked to its abundance in dietary fiber, lipids, phenolic compounds, tannins, and flavonoids, including anthocyanins, flavones, and flavanones [8,9]. Incorporating whole sorghum flour into food products can provide bioactive properties, such as antioxidant, cytotoxicity, and anti-inflammatory effects [10,11,12].

Germination is a complex, effective, and inexpensive biochemical process used to improve grain quality [13,14]. During the germination process, several enzymes can be generated or activated, including proteases, amylases, β-glucanases, and phytases. This leads to the breakdown of starch, which is used as an energy reserve for the germination process. As a result, elevated levels of reducing sugars, soluble fibers, free amino acids, oligopeptides, and mono- and oligosaccharides are produced [15,16].

Increasing the germination time can induce modifications in the thermophysical properties of starch, resulting in a higher degree of gelatinization, a higher gelatinization temperature, and a lower viscosity. It can also cause protein denaturation and a decrease in the surface tension of the molecules, resulting in increased foaming, emulsifying, and gelling properties of the flour [13,17].

The germination process induces biochemical changes in grains, which are significant for food processing. Previous studies have prioritized the technological and nutritional modifications of sorghum grains during germination [4,18]. However, there is a research gap for chemical and bioactive transformations. The aim of this study was to evaluate the chemical and bioactive properties of sorghum in addition to the influence of germination on its technological and nutritional characteristics, as an approach to reintroducing it into human food.

## 2. Materials and Methods

### 2.1. Samples and Flour Preparation

The sorghum (*Sorghum bicolor* (L.) Moench) seeds were provided by the company NHD-Foods (Uberaba, Minas Gerais, Brazil). In this work, two types of sorghum flour were studied: native sorghum flour (without any germination process) and sprouted sorghum flour, both from the same batch. The process of germination of sorghum grains followed the specifications described by Contreras-Jiménez [19] and Ocheme [15], with slight modifications. The sample was manually cleaned and sorted to remove foreign material and defective grains. After sanitization, the grains were forwarded to the incubator (incubator type B.O.D, CE-300/350-FAU, CIENLAB) and 1 kg of sorghum grains was immersed in 2.5 L of distilled water for 24 h at 21 °C, with water exchange performed every 12 h. After incubation, the water was drained and the sorghum grains were dispersed on cotton bags then placed back in the incubator at 30 °C for 24 h. After germination, the grains were dried in an oven (Model CE-205/100, CIENLAB, São Paulo, Brazil) at 40 °C for 12 h.

The native and sprouted sorghum grains were ground in a mill (SOLAB, SL31, São Paulo, Brazil) equipped with a 0.25 mm sieve. The flours obtained from both samples were packed in polypropylene bags, coded, and stored at room temperature.

### 2.2. Thermo-Mechanical Properties of the Flours

#### 2.2.1. Granulometry

The particle size distributions of both native and sprouted sorghum flour were determined using official method N°. 66–20 of the American Association of Cereal Chemists (AACC) [20]. In this procedure, approximately 100 g of each flour was subjected to sieving with agitation for 15 min using a sieve shaker from Bertel Indústria Metalúrgica Ltd.a., Caieiras, São Paulo, Brazil. A set of sieves with 20, 30, 50, 60, and 100 “Mesh Tyler” (with apertures of 850, 600, 300, 250, and 150 µm, respectively) and a base were employed. The weight of flour retained on each sieve and the base was measured and expressed as a percentage (%).

#### 2.2.2. Color Parameters

The color parameters of native and sprouted sorghum flours were determined using a colorimeter (model CR400, Konica Minolta, NJ, USA) with an integrating sphere and a 45° viewing angle (illumination d/45 and illuminant D). The illuminance values were determined on the surface of the samples at 3 different points by the luminosity parameters (L*), green–red component (a*), yellow–blue component (b*), saturation (c*), and hue angle (h*).

#### 2.2.3. Flour Granules Microscopy

The microstructures of the native and sprouted sorghum flours were determined following the methodology described by Guerra-Oliveira [21]. A DM750 microscope (Leica Microsystems, Wetzlar, Germany) at 20× magnification combined with LAS-EZ 3.0 software (Leica Microsystems, Wetzlar, Germany) was used to capture the images.

#### 2.2.4. Flour Gel Hydration Properties

The water absorption capacity (WAC) and oil absorption capacity (OAC) were determined according to the methodology described by Beuchat [22], Köhn [23], and Lin [24], respectively. The determination of the swelling power (SP) and water solubility index (WSI) of sprouted and native sorghum flours was performed as described by Spier [25]. The WAC results were reported as the ratio of the mass of water absorbed (in grams) to the initial mass of the sample (in grams). Similarly, OAC results were reported as the mean ± standard deviation of the ratio of absorbed oil mass (in grams) to initial sample mass (in grams). Solubility was determined by calculating the ratio of soluble mass to initial mass, expressed as a percentage (%). The swelling power was determined by expressing the ratio of the final mass (g) to the initial mass of the sample (g).

#### 2.2.5. Pasting Properties

The apparent viscosity profile of the samples was assessed using a rapid visco-analyzer instrument (RVA 4500 series, Perten Instruments, USA) following the method outlined by Curti [26]. Each sample of 3.5 g with a moisture content of 14% was mixed with 25 g of distilled water to form a water slurry. These slurries were then placed in aluminum canisters. The mixture was subjected to a heating process starting at 50 °C for 1 min, followed by an increase to 95 °C at a rate of 12 °C per minute. The temperature was maintained at 95 °C for 2.5 min, after which the suspension was cooled back to 50 °C at the same rate. The paddle speed was initially set at 960 rpm for the first 10 s and then maintained at 160 rpm for the duration of the test.

Thermocline software (Version 3.15, Perten Instruments, LAF Technolgies, Sydney, Australia) was utilized to derive the paste parameters. Parameters related to water–paste bonding, including pasting temperature (the temperature at which gelatinization initiates, °C), final viscosity (viscosity of the slurry at the test’s conclusion), breakdown (the disparity between peak viscosity and minimum viscosity during the holding period), and setback (the difference between peak and final viscosities), were computed from the pasting curve presented in centipoise (cp).

#### 2.2.6. Firmness

To determine the firmness of the gels formed by the flour, in a thermostatic bath (Julabo, SW22; Seelbach, Germany) at 90 °C, the flours were homogenized with distilled water at a concentration of 50% (*w*/*v*) for 5 min until the gel structure was formed. Subsequently, 40 mL of each sample was placed in an acrylic container with a lid, which had been previously identified, and stored under refrigeration at 15 °C until further analysis.

The firmness of the gels were determined 24, 48, and 72 h after preparation using a texturometer (model TA HD plus, Stable Micro System, Godalming, UK) and the Exponent Lite 2016 program, version 6.1.16 lite. Compression of the gels was performed at a speed of 2 mm/s and 15 mm with a 1.0 in cylindrical probe, and a 5 kg load cell. From the penetration curve, the gel firmness parameters were obtained, expressed in g/cm^2^. The strength is defined as the maximum force observed during the initial penetration cycle of the probe into the gel.

### 2.3. Nutritional Profile and Chemical Composition

#### 2.3.1. Moisture and Nutritional Characterization

The moisture content was determined using the official method of analysis (AOAC) N°. 925.45b [27] in an electronic moisture balance (ADAM, PMB 163, Oxford, MI, USA). Ash content was determined by carbonization, as described by the official method of analysis N°. 935.42 [27]. The determination of the total fat content was performed as described by the official method of analysis N°. 989.05 [27]. The protein content was determined using the macro-Kjeldahl method described by the official method of analysis N°. 991.02 [27], using a nitrogen to protein conversion factor of 6.25. Total dietary fiber content (soluble and insoluble) was determined by following official methods of analysis N°. 991.43 and 992.16 [27]. The carbohydrate content was estimated as the difference between 100% and the sum of the percentages obtained in the analysis of moisture, ash, fiber, protein, and lipids. Energy contribution was calculated as described by Regulation (EU) N°. 1169 [28].

#### 2.3.2. Free Sugars

The analysis of free sugars was conducted following the approach outlined by Barros [29] using high-performance liquid chromatography coupled with a refractive index detector (HPLC-RI, Knauer, Smartline system 1000, Berlin, Germany). The identification of sugars was made by comparing the retention times of the sample peaks with authentic standards and the quantification was performed by internal normalization of the chromatographic peak area using melezitose as an internal standard. The results were expressed in g per 100 g dry weight of the sample.

#### 2.3.3. Organic Acids

The analysis of organic acids was performed using ultra-fast liquid chromatography coupled to a diode array detector (UFLC-DAD; Shimadzu Cooperation, Kyoto, Japan) according to the procedure described by Barros [29]. The identification of organic acids and their quantification was achieved by the comparison of retention times and spectra with commercial standards and respective calibration curves. The results were expressed in g per 100 g dry weight of the sample.

#### 2.3.4. Fatty Acids

The fatty acid profile was determined by gas chromatography coupled to a flame ionization detector (GC-FID, DANI instrument model GC 1000, Milan, Italy) as described by Barros [29]. Fatty acid identification was performed based on the relative retention times of the peaks of the standard mixture of 37 FAMEs and the samples. The fatty acid outcomes were analyzed using Clarity 4.0.1.7 software (DataApex, Podohradska, Czech Republic) and presented as relative percentages.

#### 2.3.5. Tocopherols

The extraction procedure and the chromatographic characterization of tocopherols were performed according to the procedures described by Barros [29]. Data were analyzed using Clarity 2.4 software (DataApex, Prague, Czech Republic). Quantification was based on fluorescence signal response using the internal standard method and by chromatographic comparison with standard calibration curves for tocopherols. The results were expressed in g per 100 g dry weight of the sample.

### 2.4. Bioactivities and Phenolic Profile

#### 2.4.1. Extract Preparation

For analysis of the bioactivity and phenolic compounds of the flour samples, hydroethanolic extracts were prepared. For each extract, 1 g of sample was weighed and subjected to a maceration process with an ethanol/water solution (80:20, *v*/*v*; 30 mL) at room temperature under constant magnetic stirring (150 rpm) for 1 h. Subsequently, the solution was filtered through a filter paper (Whatman no. 4; Sigma-Aldrich, St. Louis, MO, USA) and the process was repeated using the same volume of the hydroethanolic solution and stirring time. Finally, the alcoholic fraction of the obtained extract was evaporated at reduced pressure (Büchi R-210, Flawil, Switzerland) and the aqueous fraction obtained was frozen and subsequently lyophilized (47 °C, 0.045 bar; FreeZone 4.5, Labconco, Kansas City, MO, USA).

#### 2.4.2. In Vitro Antioxidant Activity

The assessment of antioxidant potential using the TBARS assay followed the procedure outlined by Souilem [30]. A cell-based in vitro method was employed to evaluate the ability of the samples to inhibit the generation of thiobarbituric acid reactive substances (TBARS) in porcine brain homogenates (Sus scrofa). The results were quantified in terms of IC_50_ values, representing the concentration of the extract that yields 50% antioxidant activity (µg/mL). Additionally, the antihemolytic activity of the hydroethanolic extracts was appraised using another cell-based assay, OxHLIA, as previously detailed by Lockowandt [31]. The results were also expressed as IC_50_ values (µg/mL), which correspond to the concentration of each extract that provides a 60-min delay in cell hemolysis (∆t). In both experiments, Trolox served as the positive control.

#### 2.4.3. In Vitro Antiproliferative Activity

For the evaluation of the cytotoxic potential of the hydroethanolic extracts of the native and sprouted sorghum flours, the sulforhodamine B (SRB) assay previously described by Mandim [32] was performed. Four tumor cell lines were used: AGS (gastric adenocarcinoma), CaCo-2 (colorectal adenocarcinoma), MCF-7 (breast adenocarcinoma), and NCI-H460 (lung carcinoma). Non-tumor cell lines were also tested: Vero (African green monkey kidney) and PLP2 (primary porcine liver cell culture). The results were expressed as the concentration of extract with the ability to inhibit cell proliferatCion by 50%, GI_50_, using ellipticine as positive control.

#### 2.4.4. In Vitro Anti-Inflammatory Activity

The anti-inflammatory potential of each sample was evaluated through the production of nitric oxide by lipopolysaccharide-stimulation of a mouse macrophage cell line (RAW 264.7) obtained from the DMSMZ-Leibniz-Institut DSMZ-Deutsche Sammlung von Mikroorganismen und Zellkulturen GmbH [14]. Nitric oxide quantification was performed using a Griess reagent system kit (nitrophenamide, ethylenediamine, and nitrite solutions) using a nitrite calibration curve (100 mM sodium nitrite at 1.6 mM) prepared on a 96-well plate. The amount of nitric oxide produced was determined by measuring absorbance at 540 nm (Synergy H1, BioTek Instruments, Winooski, VT, USA) for each sample and comparing this with the calibration line of the standard (*y* = 0.0068*x* + 0.0951, *R*^2^ = 0.9864). The results were determined by graphically representing the percentage of nitric oxide-production inhibition against the concentration of the extract. The concentration causing 50% inhibition of nitric oxide production, known as IC_50_, was quantified. Dexamethasone was used as a positive control.

#### 2.4.5. Phenolic Profile

The phenolic profile was evaluated through chromatographic analysis according to the procedure described by the authors [33], in which a Dionex Ultimate 3000 UPLC HPLC instrument (Thermo Scientific, San Jose, CA, USA) was used, composed of a quaternary pump and with double on-line detection: a diode array detector (DAD) selecting wavelengths 280 nm and 370 nm, and, in sequence, a mass spectrometry (MS) detector. A Waters Spherisorb S3 ODS-2 C18 column (3 μm, 150 × 4.6 mm, Watersm Milford, MA, USA) was used for sample separation at an operating temperature of 35 °C. The mobile phase was 0.1% formic acid in water (A) and acetonitrile (B). The elution gradient was 15% B (5 min), 15% B to 20% B (5 min), 20–25% B (10 min), 25–35% B (10 min), and 35–50% B (10 min), and the column was rebalanced (10 min) using a flow rate of 0.5 mL/min.

MS detection was performed using an Ion Trap Linear LTQ XL mass spectrometer (ThermoFinnigan, San Jose, CA, USA) with an ESI electrospray ionization source. Nitrogen was used as the carrier gas at 50 psi. The system operated with a spray voltage of 5 kV at 325 °C with a capillary voltage of −20 V. A tube lens offset voltage of −66 V was maintained. Spectra were recorded in negative ion mode between 100 and 1500 m/z. The collision energy used was 35 (arbitrary units).

The results were analyzed using the Xcalibur^®^ program (ThermoFinnigan, San Jose, CA, USA). For the identification of phenolic compounds, these results were compared with retention times in the literature, and/or when possible, to the UV–Vis mass spectra. Quantitative analysis was performed using 7-level calibration straight lines for each standard: caffeic acid (*y* = 388,345*x* + 406,369, *R*^2^ = 0.9998; LOD (Limit of Detection) = 0.78 μg/mL; LOQ (Limit of Quantification) = 1.97 μg/mL); chlorogenic acid (*y* = 168,823*x* − 161,172, *R*^2^ = 0.9998; LOD = 0.20 µg/mL; LOQ = 0.68 µg/mL); protocatechuic acid (*y* = 214,168*x* + 27,102, *R*^2^ = 0.9999; LOD = 0.14 μg/mL; LOQ = 0.52 μg/mL); apigenin-6-*C*-glucoside (*y* = 197,337*x* + 30,036, *R*^2^ = 0.9999; LOD = 0.19 µg/mL; LOQ = 0.63 µg/mL); (-)-catechin (*y* = 84.950*x* − 23.200, *R*^2^ = 0.999; LOD = 0.17 μg/mL; LOQ = 0.68 μg/mL); naringenin (*y* = 18,433*x* + 78,903, *R*^2^ = 0.9998; LOD = 0.17 µg/mL; LOQ = 0.81 µg/mL); and quercetin-3-*O*-glucoside (y = 34,843x − 160,173, LOD = 0.21 µg/mL; LOQ = 0.71 µg/mL); quantification was based on the UV–Vis signal of the commercial standards at their maximum wavelength and, when not available, from other compounds within the same phenolic group. The results were expressed in mg per g extract.

### 2.5. Statistical Analysis

All experiments were conducted thrice and the mean value expressed as mean ± standard deviation (SD). IBM SPSS Statistics for Windows, Version 23.0 (IBM Corp., Armonk, New York, NY, USA) was employed to test for significant differences between samples using the Student’s t-test with a 95% significance level.

## 3. Results and Discussion

### 3.1. Thermo-Mechanical Properties of the Flours

Table 1 presents the physical properties of both native and sprouted sorghum flours. The flour color has a major impact on the acceptability of bakery products to consumers. The use of whole grain flours results in darker colors because of the high fiber content present in whole grains, which provides a better nutritional profile with health benefits, a fact that has a direct impact on consumer preferences [34].

Flour-color variations significantly influence bakery products’ sensory attributes. Lighter flours yield softer textures and lighter crusts, while darker counterparts contribute nuttier flavors and firmer textures due to heightened protein content. Aromatic compounds formed during the Maillard reaction in darker flours impact overall scent. Flour color also reflects nutritional differences, with darker flours often signifying higher fiber and nutrient content. Consumer preferences vary, influencing product perception. Consistent flour color is crucial for maintaining product-appearance uniformity. Understanding these effects aids bakers in selecting flours to achieve desired sensory qualities in their baked goods [35,36,37].

The germination of sorghum grains changed the color of the flour in the L* and a* parameters. This is related to the increase in some compounds during germination, such as β-carotene and some tannins, found in the pericarp of grains [38,39]. Studies assessing effects of the malting and fermentation of cereals [39] reported that increased times for these two steps also increased the brightness of the flour. This is consistent with the results of the current study, which observed a light flour with a tendency towards yellow.

In the baking process, flour particle size plays a critical role: smaller particle sizes increase the amount of accessible starch, promoting high levels of gelatinization and retrogradation, contributing to dough viscoelastic properties as well as final product volume and firmness [26].

The flour’s particle size distribution is determined by the milling characteristics and grain properties. From the gravimetric analysis of the flour, it was possible to observe that more than 74% of the particles were retained in the sieves with aperture between 0.500 µm and 250 µm. Martino [40] analyzed the particle sizes of different sorghum flours and obtained similar results, with higher retention of the flours in the 0.42 µm aperture sieve. The variation in particle size could be attributed to the process of soaking the grains to facilitate germination, which in turn makes them easier to grind, leading to a finer flour [41].

Germination did not significantly affect water absorption capacity (WAC), whereas oil absorption capacity (OAC) increased approximately 8% in the germinated sorghum flour compared to the native sorghum flour. Similar values were reported by Rothschild [42], as they demonstrated that germination of quinoa grains did not cause significant differences in WAC before 48 h of germination, while OAC increased after 24 h. The enhanced oil absorbency is linked to the solubilization and dissociation of proteins, which increases the surface availability of lipophilic proteins. The hydrophobic amino acids bind to the hydrocarbon chain of the oil, resulting in the improvement of flours made from sprouted grains [15].

The SP refers to the ability of the starch granules to swell during heating and with water excess. As it is possible to observe from the results obtained, the sorghum germination process caused a decreased swelling power, with the native sorghum flour presenting a value of 7.64 ± 0.05 g/g while the sprouted sorghum flour presented 6.59 ± 0.05 g/g. However, the WSI increased with the germination process. The changes in SP and WSI are attributed to changes in the starch structure, primarily due to an increase in the amylose/amylopectin ratio, as a result of enzymatic activity occurring during grain germination that selectively hydrolyzes amylopectin chains [4]. Germination also leads to the formation of dextrins, oligosaccharides, and fermentable sugars that have no SP and interfere with starch by forming more compact gels, therefore creating flours with lower SP [17].

Starch gel firmness is a result of starch retrogradation, which depends on factors such as flour particle size and amylopectin crystallinity [17]. The firmness of the gels of native sorghum flour were higher than those of sprouted sorghum, with values of 220 ± 11 and 23.7 ± 0.8 g/cm^2^, respectively. The firmness of the gels of sprouted sorghum flour reached 23.7 ± 0.8 g/cm^2^ in the first 24 h and increased to 52.6 ± 0.1 g/cm^2^ after 72 h of preparation. The native sorghum flours varied from 220 ± 11 (24 h) to 307 ± 24 g/cm^2^ (72 h), so the germination was prolonged to decrease the retrogradation and syneresis of sorghum starch gels. This phenomenon is observed because during germination there is an activation of various amylolytic enzymes, such as amylases, β-glucanases, and of proteases, which further degrade the outermost branches of amylopectin in the starch granule [17].

**Figure 1:**
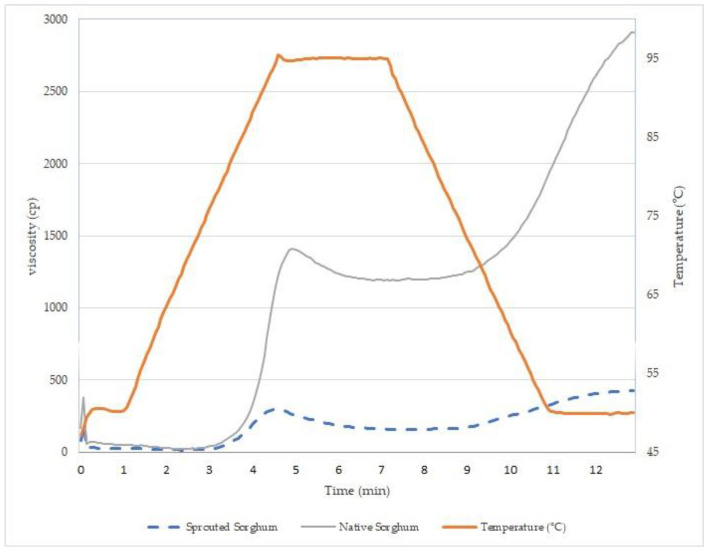
Sorghum flour sample viscosity properties.

The paste profile in water was affected by sorghum grain germination (Figure 1). The germination process caused a decrease in viscosity during the heating and cooling stages. The paste temperature for the native sorghum flour was 86.6 °C, while for the sprouted sorghum flour it was 84.9 °C, indicating that germination affects the temperature needed to achieve intumescence (dilatation) of the starch granule. A decrease in peak viscosity was also observed after the grain germination process, as well as an increased gelatinization temperature. This may be due to many factors, including the degradation of starch into low-molecule-weight particles, the activity of the enzyme α-amylase, and, finally, changes in proteins and fatty acids during the germination process [41].

The setback value reflects the tendency for the starch to retrograde in the paste. This value is lower for the germinated sorghum sample, suggesting a decrease in the retrogradation capacity of the starch compared to that of native sorghum, confirming the data on the starch gel firmness. During germination, hydrolysis of the outermost part of the amylopectin by α-amylase occurs, and, consequently, it is no longer possible for large amylopectin crystals to form; the remaining smaller crystals are not sufficient to promote a large increase in viscosity during the cooling of the paste [4].

The firmness and pasting properties of germinated sorghum flour can significantly affect the texture and sensory characteristics of final products, especially in bread and cakes where viscosity is crucial for quality. Careful consideration is recommended when using this flour in products that do not heavily rely on viscosity, such as biscuits [43].

### 3.2. Nutritional Profiles and Chemical Compositions of the Flours

Table 2 presents the nutritional profiles and energy contributions of native and sprouted sorghum flours. The carbohydrate contents between the flours were not significantly different (*p* = 0.05). However, for the remaining parameters there were significant differences. The flour of sprouted sorghum showed higher values of moisture, protein, and ash, while the native sorghum flour showed higher contents of total fat and total dietary fiber.

The native sorghum flour showed a lower moisture content (9.505 ± 0.005 g/100 g fw) than the sprouted sorghum flour (10.79 ± 0.01 g/100 g fw). These differences in moisture content may stem from the germination process, as the grain undergoes a hydration process that stimulates the biochemical processes inherent in the germination process. The sprouted sorghum flour has a slightly higher ash content (1.03 ± 0.01 g/100 g dw) than the native sorghum flour (0.89 ± 0.01 g/100 g dw).

The germination process significantly increased the protein content, as the native sorghum flour showed values of 5.9 ± 0.1 g/100 g dw, while the sprouted sorghum presented around 9.03 ± 0.42 g/100 g dw. Xu [14] has already described an increase in protein content during sprouting, which may be caused by the synthesis of enzymes by sprouting seeds, alteration of composition as a result of breakdown of other components, and the synthesis of new proteins formed during sprouting.

Regarding total fat, the sprouted sorghum flour showed lower amounts when compared to the native sorghum flour, with values of 3.018 ± 0.003 and 4.21 ± 0.09 g/100 g dw, respectively. In the germination process, there is an increase in the activities of lipolytic enzymes, which hydrolyze the fat component into fatty acids and glycerol, which results in a decrease in fat content [17]. In conjunction, it may have been influenced by the development of β-oxidation and the glyoxylate cycle, which are used as energy sources for grain germination [44].

Finally, the energy contributions obtained was 417.5 ± 0.4 kcal/100 g dw for the native sorghum flour and 384 ± 1 kcal/100 g dw for the sprouted sorghum flour. This difference in energy value between the flours is mainly due to the biochemical and physiological changes that occur during germination to provide energy for new plant growth [17].

**Figure 2:**
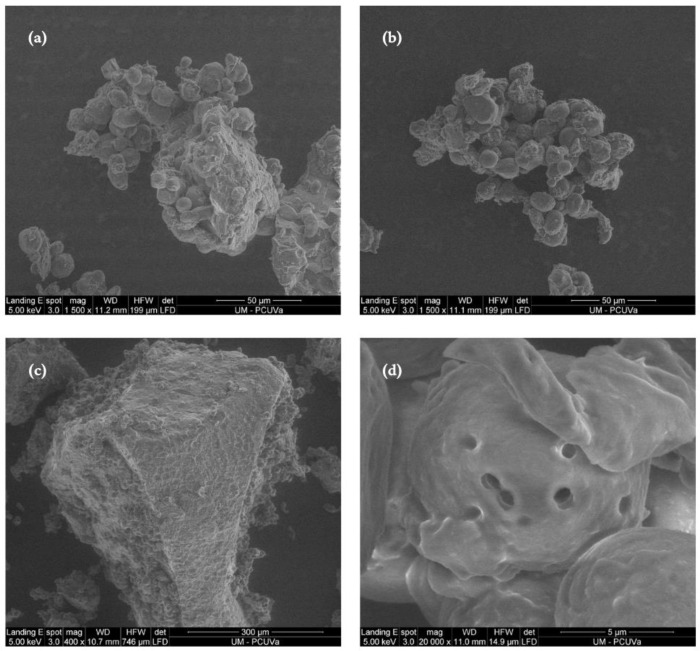
Scanning electron micrographs of native and sprouted sorghum flour: (**a**) native sample (1500×), (**b**) sprouted sample (1500×); (**c**) native sample (12,000×); and (**d**) sprouted sample (12,000×).

Although the native and sprouted sorghum flours do not present significant differences in carbohydrate content, the germination process caused several biochemical changes as shown in the scanning electron micrograph illustrated in Figure 2. The process of germination caused alterations to the surface of starch granules, resulting in their fragmentation and the formation of enlarged pores on their surfaces (Figure 2a,c), features that are not present in the starch granules of the native sorghum grains (Figure 2b,d). The changes observed in sprouted sorghum micrographs, such as alterations in surface area and the emergence of increased pores, could be linked to the enzymatic degradation of amylopectin molecules. These molecules break down into oligosaccharides that may serve as a source of energy for plant growth [41].

The breakdown of starch granules during germination is caused by the ratio of amylose and amylopectin, which significantly affects the morphology of the granules [4]. Sorghum starch has a higher proportion of short amylopectin chains, which can result in a more porous granular structure that is more vulnerable to enzymatic degradation than regular starch [45].

### 3.3. Chemical Compositions of the Flours

The results related to free sugars and organic acids of the native and sprouted sorghum flours are described in Table 3. Regarding free sugars, glucose and sucrose were identified and quantified in the sprouted sorghum flour, while in the native sorghum flour only sucrose was detected. This sugar content did not significantly change with the germination of the grain, which remained at 0.75 g/100 g dw for both flours. With the contribution of glucose in the germinated grains, the total free sugar content was of 0.82 ± 0.04 g/100 g dw and 1.49 ± 0.04 g/100 g ps for the native and sprouted sorghum flours, respectively.

Plant sugars are a form of energy for the germination process of the grain, so during germination there is the activation of amylolytic enzymes, which hydrolyze the outermost part of the starch granules and form lower-molecular-weight sugars, and the sprouts use these as energy sources to promote their growth [16]. An increase in the total sugar content was observed in germinating varieties of buckwheat and quinoa, where it is worthwhile to point out that only after 12 h of germination were significant changes observed [46,47].

Regarding organic acids, a total of five compounds were detected, namely oxalic, malic, citric, succinic, and fumaric acids. In the sprouted sorghum flour, an increase in total organic acid content was observed (1.75 ± 0.01 g/100 g dw), with malic acid mainly responsible for this increase (0.11 ± 0.01 g/100 g dw and 1.49 ± 0.01 g/100 g dw, in the native and sprouted sorghum flours, respectively). In contrast, germination significantly reduced the amount of fumaric acid, which showed only traces in the germinated sorghum flour, while in the native sorghum flour a concentration of 0.67 ± 0.01 g/100 g dw was found. Plants generate organic acids through the incomplete oxidation of photosynthetic products. Within germination processes, these are synthesized by the glyoxylate cycle [48]. Organic acids, in addition to intervening in the synthesis of amino acids in cereals during germination, also help to acidify the endosperm to assist in the process of starch degradation, which is used later as an energy source [44,49].

In studies on the concentration of organic acids in barley seeds during germination, it was observed that malic acid was the most abundant organic acid during the first three days of germination, followed by citrate and then fumaric acid [44], which is in agreement with the observations of the present study. Malic acid is the most commonly found organic acid in plant tissues, and it fulfills many functions in plant metabolism such as photosynthesis, maintenance of internal pH, and the transport and exchange of reducing equivalents between cellular compartments [48].

Organic acids have been found to exhibit antioxidant activity and inhibit the growth of microorganisms. Additionally, they offer sensory benefits in bakery products. Studies have shown that the use of organic acids, such as citric and malic acid, promotes yeast activity, resulting in bread with greater volume, lower moisture content, and an extended shelf life [50,51].

The germination process decreased the content percentage of palmitic and oleic acids by around 14% (Table 4). However, there was a significant increase of approximately 36% in linoleic acid content. A change in fatty acid profile during the germination process was also observed in studies of flaxseed germination, indicating that germination increased the proportion of unsaturated fatty acids in flaxseed [52]. Fatty acids stored in grain lipids contain high concentrations of glyoxalate cycle enzymes, which allows for the conversion of stored fatty acids into carbohydrates during germination [49]. The total fat content in flour can influence its stability and shelf life. Fats in flour can undergo oxidation, leading to rancidity and off-flavors. Therefore, a decrease in total fat content can contribute to improved flour stability [53]. Reducing fat levels in flour can also result in a longer shelf life for the flour itself and the products made from it. Rancidity caused by fat oxidation not only affects the taste and aroma of the flour but can also negatively impact the quality of baked goods produced with that flour [54]. However, it is important to note that fats in flour can also play a role in the texture, flavor, and moisture retention of baked products. Therefore, any adjustments to fat content should consider the desired qualities of the final product [55].

Tocopherols, also known as vitamin E, are important antioxidant compounds naturally present in many plant-based foods [56]. Both sorghum flours showed α-tocopherol and γ-tocopherol (Table 4). However, the sprouted sorghum flour showed lower tocopherol concentration, especially for γ-tocopherol, which decreased to 0.234 ± 0.003 g/100 g dw.

In a study of 97 sorghum genotypes, researchers investigated the prevalence of tocopherols and found that the tocopherol profile varies significantly, similar to the genetic diversity of the sorghum. The mechanisms responsible for modifying the concentration and profile of vitamin E in sprouted sorghum are not yet fully understood. Research on the stability of vitamins during sorghum germination has shown a decrease in overall tocopherol concentration, particularly for γ-tocopherol, while β-tocopherol increased. This indicates that some of these compounds may be utilized to meet nutritional requirements during grain development [56].

Finally, regarding the phenolic composition of hydroethanolic extracts from native and sprouted sorghum samples, Table 5 presents the chromatographic information, including retention time, λmax in the visible region, molecular ion, and main MS^2^ fragments, along with the tentative identification and quantification (mg/g extract) obtained by HPLC-DAD-ESI/MS. Within the sorghum samples, 21 phenolic compounds were tentatively identified, including 5 phenolic acids, 7 pyrano-flavanone-flavanols dimers, 4 *C*-glycosylated flavones, 3 flavanones, and 2 flavonols. The phenolic-compound profile coincides with that previously published by other authors, so the attempted identification of peaks was based on a comparison with data from the literature [12,57,58,59,60,61].

The profiles of both samples, in qualitative terms, are quite similar, except for the absence of protocatechuic acid in the unprocessed sorghum grains. In quantitative terms, it is in the native sorghum sample that a higher concentration of total phenols is found (15.53 ± 0.01 mg/g extract), mainly due to the presence of flavanone compounds, specifically *O*-glycosylated naringenin derivatives that represent 30% of the total phenolic compounds in this sample, followed by C-glycosylated apigenin derivatives, which represent 20%. For the sprouted sorghum sample, total phenolic-compound values are four times lower than in the unprocessed samples (3.72 ± 0.02 mg/g extract). In the samples of sprouted sorghum, the majority of phenolic compounds are phenolic acids, of which caffeoylglycerol stands out.

The composition of phenolic compounds in plants and cereals varies depending on their genotype, and previous studies show that brown and black genotypes naturally have higher concentrations of phenolic compounds than red and white sorghum genotypes [62]. In addition to the genetic factor of sorghum grains affecting phenolic composition, other studies show that deficient irrigation significantly affects the concentration of these types of phenolic compounds and their antioxidant activities [60].

Studies performed on rice, buckwheat, and flaxseed have shown an increase in phenolic compounds after germination [47,52,63]. The increase in phenolic compounds can be explained by the enzymatic synthesis that occurs during germination through the phenylpropanoid pathway, as well as the hydrolysis of cell wall polysaccharides, which releases phenolics that were previously bound within the cell wall [63]. However, in the present work, no such trend was observed, so further studies will be needed to ascertain the cause of this incongruence. One hypothesis for these results may be that many of these phenolic compounds are highly glycosylated and, during germination, the glycosidic bond is broken to provide sugar to the germinating system.

### 3.4. Bioactivities Profiles of Hydroethanolic Extracts from Native and Sprouted Sorghum Flours 

On the TBARS assay, the native sorghum flour presented an IC_50_ value of 10.4 ± 0.9 μg/mL, which was higher than that of trolox (3.7 ± 0.5 μg/mL), which was used as a positive control. In contrast, none of the flours showed antihemolytic activity in the OxHLIA assay.

However, there are studies described in the literature reporting the antioxidant activity of native sorghum flour from other types of in vitro tests, such as those using the DPPH (2,2-diphenyl-1-picrylhydrazyl) radical, 3-ethylbenzothiazoline-6-sulfonic diammonium salt (ABTS), and the reduction of iron (FRAP) [62]. Studies on the antioxidant capacity of six sorghum genotypes showed that antioxidant activity is mainly related to phenolic concentration, with the brown and black sorghum varieties (with high concentrations of total phenolics, and rich in 3-deoxyanthocyanidins and condensed tannins) showing higher antioxidant activities than the red and white sorghum varieties [61].

The EC_50_ value of sprouted sorghum (1.9 ± 0.4 μg/mL, TBARS) was almost twofold higher than that of the positive control (trolox, 3.7 ± 0.5 μg/mL). In previous studies on the in vitro antioxidant activity of sprouted brown rice and buckwheat, a higher antioxidant capacity was also observed, as hydrolytic enzymes can release free phenolic compounds with more effective antioxidant activity [47]. Furthermore, germination at high temperature (42 °C) induces several radical-scavenging enzymes, such as superoxide dismutases, glutathione S-transferases, catalases, peroxidases, and enzymes in the ascorbate–glutathione cycle that maintain a balance of redox homeostasis [63].

The hydroethanolic extracts of the native and sprouted sorghum flours showed no cytotoxicity or anti-inflammatory activity at the maximum concentration tested (400 μg/mL). Despite not revealing antitumor activity (in AGS, CaCo-2, MCF7, and NCI-H460 cell lines), the results obtained allow us to conclude that the flours are not toxic to non-tumor cell lines (PLP2 and VERO) and, therefore, can be used for human food without risks to consumers’ health.

## 4. Conclusions

Germination is a very complex biochemical process that is a natural, simple, and economical way to improve the nutritional quality of grains. Regarding the physical characteristics of the flours, it was observed that germination had significant impacts, namely giving smaller flour size, lighter coloration (flours with a tendency to yellow), higher firmness of the gels, increased WSI and OAC, and decreased SP.

For the nutritional characterization of the native and sprouted sorghum grain flours, the germination significantly affected macronutrients, with a higher percentage of moisture being observed in the sprouted sorghum, as well as higher ash and protein contents. On the other hand, a decrease in fat and dietary fiber contents was observed, which may indicate that these have been consumed during the germination process. The energy contribution of the sprouted sorghum flours was also decreased compared to native grains, which may be due to the breakdown of carbohydrates as a source of energy for the germination process, as for fat and fiber contents.

The germination process of sorghum grain also changed its chemical characteristics. Starch degradation was observed in the electron micrographs of native and germinated starch granules and by the increase in lower-molecular-weight molecules, which led to an increase in the content of free sugars and organic acids in the sprouted sorghum.

The sprouted sorghum exhibited a concentration of polyunsaturated fatty acids nearly seven times higher than that of native sorghum, which can be primarily attributed to the presence of linoleic acid (C18:2n6c, 41.907 ± 0.037%). The opposite effect was verified for tocopherols and phenolic compounds, where germination precisely decreased the concentrations of these types of compounds.

Finally, regarding the bioactive properties of the developed flours, it was again with the germinated sorghum grain flour that the best results were obtained for lipid-peroxidation-inhibition capacity. However, it showed no effect on the inhibition of oxidative hemolysis in erythrocytes. Regarding cytotoxicity for non-tumor cell lines, the native and sprouted sorghum flours showed no activity up to the maximum concentration tested (400 μg/mL), which reveals their safety for use in food for consumption.

## Figures and Tables

**Table 1:** Parameters of color, granulometry, WAC, OAC, SP, WSI, firmness, and pasting properties of the native and sprouted sorghum flours (mean ± SD).

	Sprouted Sorghum	Native Sorghum	*p*-Value ^1^
Color parameters			
L*	73.5 ± 0.3	76.7 ± 0.5	<0.001
a*	0.92 ± 0.07	0.24 ± 0.01	<0.001
b*	20.4 ± 0.4	21.4 ± 0.7	0.001
Granulometry			
Sieves/Opening (µm)	Retained Sample (%)		
16/1.18	0.16 ± 0.05	0.42 ± 0.02	<0.001
20/85	7.9 ± 0.9	7.1 ± 0.2	0.002
35/5	57 ± 2	78.6 ± 0.8	<0.001
60/250	17 ± 3	10.2 ± 0.9	<0.001
Base/<150	17.5 ± 0.3	3.7 ± 0.4	<0.001
WAC (g/g)	2.349 ± 0.001	2.35 ± 0.03	0.793
OAC (g/g)	1.91 ± 0.02	1.77 ± 0.01	<0.001
SP (g/g)	6.59 ± 0.05	7.64 ± 0.05	0.004
WSI (%)	0.89 ± 0.02	0.53 ± 0.02	<0.001
Firmness (g/cm^2^)			
24 h	23.7 ± 0.8	220 ± 11	<0.001
48 h	40 ± 6	229.024 ± 0.008	<0.001
72 h	52.6 ± 0.1	307 ± 24	<0.001

WAC: water absorption capacity; OAC: oil absorption capacity; swelling power (SP); water solubility index (WSI). Results expressed as mean ± standard deviation. ^1^ Student’s *t*-test.

**Table 2:** Nutritional profile and energy contribution of the native and sprouted sorghum flours (mean ± SD).

	Sprouted Sorghum	Native Sorghum	*p*-Value ^1^
Moisture (g/100 g fw)	10.79 ± 0.01	9.505 ± 0.005	<0.001
Nutritional profile (g/100 g dw)			
Ash	1.03 ± 0.02	0.89 ± 0.02	<0.001
Fat	3.018 ± 0.004	4.21 ± 0.09	<0.001
Protein	9.0 ± 0.4	5.9 ± 0.1	<0.001
Total dietary fiber	13.6 ± 0.6	14.9 ± 0.2	<0.001
Carbohydrate	73 ± 1	74.1 ± 0.5	0.005
Energy contribution (kcal/100 g dw)	384 ± 1	417.5 ± 0.4	<0.001

dw—dry weight basis; fw—fresh weight. Results expressed as mean ± standard deviation. ^1^ Student’s *t*-test.

**Table 3:** Soluble sugar and organic acid contributions of the native and sprouted sorghum flours (mean ± SD).

	Sprouted Sorghum	Native Sorghum	*p*-Value ^1^
Soluble sugars (g/100 g dw)			
Glucose	0.64 ± 0.01 ^a^	nd	<0.001
Sucrose	0.84 ± 0.03 ^a^	0.82 ± 0.04 ^b^	0.094
Sum	1.49 ± 0.04 ^a^	0.82 ± 0.04 ^b^	<0.001
Organic acids (g/100 g dw)			
Oxalic acid	tr	tr	<0.001
Malic acid	1.49 ± 0.01	0.11 ± 0.01	<0.001
Citric acid	0.26 ± 0.01	0.23 ± 0.01	<0.001
Succinic acid	nd	tr	<0.001
Fumaric acid	tr	0.67 ± 0.01	<0.001
Sum	1.75 ± 0.01	0.99 ± 0.01	<0.001

dw—dry weight basis; fw—fresh weigh; tr—traces; nd—not detected; Calibration curves for sugars and organic acids were as follows: glucose (*y* = 0.935*x*. *R*^2^ = 0.999; LOD (Limit of Detection) = 0.08 mg/mL; LOQ (Limit of Quantification) = 0.25 mg/mL); sucrose (*y* = 0.977*x*, *R*^2^ = 0.999; LOD = 0.06 mg/mL; LOQ = 0.21 mg/mL); oxalic acid (*y* = 1 × 10^7^*x* + 231,891; *R*^2^ = 0.9999; LOD = 6.3 μg/mL; LOQ = 20.8 μg/mL); malic acid (*y* = 950,041*x* + 6255.6; *R*^2^ = 0.9999; LOD = 15.9 μg/mL; LOQ = 52.9 μg/mL); citric acid (*y* = 1 × 10^6^x − 10,277; *R*^2^ = 0.9997; LOD = 4.4 μg/mL; LOQ = 14.5 μg/mL); and e fumaric acid (*y* = 154,862x + 1 × 10^6^; *R*^2^ = 0.9977; LOD = 42.5 μg/mL; LOQ = 141.7 μg/mL). Results expressed as mean ± standard deviation. ^1^ Student’s *t*-test.

**Table 4:** Fatty acid and tocopherol contributions of the native and sprouted sorghum flours (mean ± SD).

Fatty Acids (Relative Percentage, %)	Sprouted Sorghum	Native Sorghum	*p*-Value ^1^
Palmitic acid (C16:0)	14.4 ± 0.1	28.019 ± 0.008	<0.001
Caprylic Acid (C8:0)	0.082 ± 0.001	0.47 ± 0.02	<0.001
Capric Acid (C10:0)	0.033 ± 0.001	nd	<0.001
Undecanoic Acid (C11:0)	0.066 ± 0.001	0.66 ± 0.03	<0.001
Lauric Acid (C12:0)	0.063 ± 0.001	0.208 ± 0.005	<0.001
Tridecanoic Acid (C13:0)	0.084 ± 0.004	nd	<0.001
Myristic Acid (C14:0)	0.101 ± 0.002	0.158 ± 0.002	<0.001
Pentadecanoic Acid (C15:0)	0.061 ± 0.001	nd	<0.001
cis-10-Pentadecenoic Acid (C15:1)	0.054 ± 0.001	1.21 ± 0.01	<0.001
Palmitic Acid (C16:0)	14.35 ± 0.11	28.019 ± 0.008	<0.001
Palmitoleic Acid (C16:1)	0.397 ± 0.007	0.431 ± 0.006	<0.001
Heptadecanoic Acid (C17:0)	0.206 ± 0.008	5.627 ± 0.005	<0.001
Stearic Acid (C18:0)	1.533 ± 0.002	3.742 ± 0.008	<0.001
Oleic Acid (C18:1n9c)	36.99 ± 0.05	50.38 ± 0.01	<0.001
Linolelaidic Acid (C18:2n6t)	0.041 ± 0.001	nd	<0.001
Linoleic Acid (C18:2n6c)	41.907 ± 0.037	5.159 ± 0.002	<0.001
Alpha-Linolenic Acid (C18:3n3)	1.709 ± 0.006	0.145 ± 0.001	<0.001
Arachidic Acid (C20:0)	0.222 ± 0.006	1.034 ± 0.005	<0.001
cis-11-Eicosenoic Acid (C20:1)	0.441 ± 0.003	0.282 ± 0.001	<0.001
cis-11,14-Eicosadienoic Acid (C20:2)	0.765 ± 0.007	nd	<0.001
Heneicosanoic Acid (C21:0)	nd	0.282 ± 0.001	<0.001
Arachidonic Acid (C20:4n6)	0.091 ± 0.001	0.78 ± 0.01	<0.001
Behenic Acid (C22:0)	0.228 ± 0.004	0.32 ± 0.01	<0.001
cis-11,14,17-Eicosatrienoic Acid (C20:3n3)	nd	0.211 ± 0.005	<0.001
cis-13,16-Docosadienoic Acid (C22:2)	0.117 ± 0.004	0.356 ± 0.002	<0.001
Tricosanoic Acid (C23:0)	0.219 ± 0.001	0.271 ± 0.002	<0.001
Lignoceric Acid (C24:0)	0.249 ± 0.001	nd	<0.001
SFA	17.5 ± 0.1	40.518 ± 0.008	<0.001
MUFA	37.89 ± 0.06	52.83 ± 0.01	<0.001
PUFA	44.62 ± 0.05	6.65 ± 0.01	<0.001
Tocopherols (g/100 g dw)			
α-Tocopherol	0.142 ± 0.005	0.127 ± 0.001	<0.001
γ-Tocopherol	0.234 ± 0.003	0.72 ± 0.01	<0.001
Sum	0.376 ± 0.003	0.85 ± 0.01	<0.001

dw—dry weight basis; SFA—saturated fatty acids; MUFA—monounsaturated fatty acids; and PUFA—polyunsaturated fatty acids. Standard calibration curves for tocopherols were as follows: α- tocopherol (*y* = 1.295*x*, *R*^2^ = 0.991; LOD (Limit of Detection) = 18.06 ng/mL; LOQ (Limit of Quantification) = 60.20 ng/UP); β-tocopherol (*y* = 0.396*x*, *R*^2^ = 0.992; LOD = 25.82 ng/mL, LOQ = 86.07 ng/mL); and e γ- tocopherol (*y* = 0.567*x*; *R*^2^ = 0.991; LOD = 14.79 ng/mL, LOQ = 49.32 ng/mL). Results expressed as mean ± standard deviation. ^1^ Student’s *t*-test.

**Table 5:** Retention time (Rt), wavelengths of maximum absorption in the ultraviolet region (λmax), mass spectral data, tentative identification, and quantification (mg/g extract) of the phenolic compounds present in the hydroethanolic extracts of sprouted and native sorghum flours (mean ± SD).

						Quantification (mg/g Extract)	
Peak	Rt (min)	λmax (nm)	[M-H]^-^ (*m/z*)	MS^2^ (*m/z*)	Tentative Identification	Sprouted Sorghum	Native Sorghum
1	5.36	253	153	109(100)	Protocatechuic acid	0.35 ± 0.01	nd
2	7.42	324	415	253(100), 179(34), 161(46), 135(5)	1-*O*-Caffeoyl-2-*O*-glucosylglycerol	0.314 ± 0.001	0.782 ± 0.003
3	7.83	319	253	179(12), 161(5), 135(100)	*O*-Caffeoylglycerol	0.234 ± 0.002	0.64 ± 0.02
4	9.92	325	253	179(39), 161(42), 135(100)	*O*-Caffeoylglycerol	0.846 ± 0.005	2.21 ± 0.02
5	10.47	323	179	135(100)	Caffeic acid	0.047 ± 0.004	0.29 ± 0.02
6	11.38	320	449	287(100), 269(5)	Dihydrokaempferol hexoside	0.3984 ± 0.0005	0.417 ± 0.0001
7	12.8	321	449	287(100), 269(5)	Dihydrokaempferol hexoside	0.3794 ± 0.004	0.513 ± 0.002
8	13.46	327	563	545(66), 503(86), 473(100), 443(69), 383(88), 353(40)	Apigenin-*C*-pentosyl-*C*-hexoside	0.0138 ± 0.003	0.169 ± 0.002
9	13.72	323	563	545(50), 503(76), 473(79), 443(100), 383(12), 353(50)	Apigenin-*C*-pentosyl-*C*-hexoside	0.29 ± 0.01	1.62 ± 0.001
10	13.96	334	563	545(12), 503(14), 473(67), 443(100), 383(37), 353(32)	Apigenin-*C*-pentosyl-*C*-hexoside	0.27 ± 0.01	1.44 ± 0.01
11	14.27	285	883	721(34), 595(100), 567(13), 433(52), 405(10), 287(10)	Pyrano-3′,4′,5′,5,7-pentahydroxyflavanone-(3 → 4)-catechin-7-O-glucoside.	0.18 ± 0.003	0.68 ± 0.01
12	15.26	274/331	563	545(17), 503(11), 473(100), 443(47), 413(11) 383(33), 353(25), 311(4)	Apigenin-*C*-pentosyl-*C*-hexoside	tr	0.0706 ± 0.0001
13	15.97	284	433	271(100)	Naringenin-*O*-hexoside	0.024 ± 0.003	1.686 ± 0.003
14	16.53	284	851	689(28),563(100),551(55),401(89),389(5)	Pyrano-naringenin-(3 → 4)-catechin-7-*O*-glucoside I	0.063 ± 0.001	0.16 ± 0.01
15	16.83	286	867	705(100), 579(52), 449(12), 417(34), 287(10)	Pyrano-naringenin-(3 → 4)-catechin-7-*O*-glucoside I	0.067 ± 0.002	0.78 ± 0.02
16	17.06	283	433	271(100)	Naringenine-*O*-hexoside	tr	2.8122 ± 0.0003
17	17.75	285	867	705(100), 579(32), 525(5), 449(32), 417(62), 287(26)	Pyrano-naringenin-(3 → 4)-catechin-7-*O*-glucoside II	0.055 ± 0.0003	0.15 ± 0.002
18	18.18	287	867	705(100), 579(43), 525(5), 449(29), 417(49), 287(5)	Pyrano-naringenin-(3 → 4)-catechin-7-*O*-glucoside II	0.102 ± 0.003	0.267 ± 0.004
19	18.43	284	579	417(50), 271(100)	Naringenin *O*-hexosyl-deoxyhexoside	tr	0.213 ± 0.002
20	20.78	285	851	689(28), 563(100), 551(55), 401(89), 389(5)	Pyrano-naringenin-(3 → 4)-catechin-7-*O*-glucoside II	0.052 ± 0.001	0.64 ± 0.02
21	21.27	285	851	689(34), 563(100), 551(46), 401(92), 389(6)	Pyrano-naringenin-(3 → 4)-catechin-7-*O*-glucoside III	0.0373 ± 0.0004	0.146 ± 0.003
					Phenolic Acids	1.79 ± 0.01	3.93 ± 0.02
					Flavanols	0.7777 ± 0.0004	0.93 ± 0.002
					*C*-glycosylated Flavones	0.5744 ± 0.0002	3.14 ± 0.01
					Flavanones	0.024 ± 0.003	4.711 ± 0.001
					Pyrano-flavanone-flavanol Dimers	0.557 ± 0.004	2.82 ± 0.02
					Total Phenolic Compounds	3.72 ± 0.02	15.53 ± 0.01

Tr–traces; nd—not detected. Calibration curves used for quantification were as follows: Caffeic acid (y = 388,345x + 406,369, LD = 0.78 μg/mL; LQ = 1.97 μg/mL, peak 5); chlorogenic acid (y = 168,823x − 161,172, LD = 0.20 μg/mL; LQ = 0.68 μg/mL, peaks 2, 3 and 4); protocatechuic acid (y = 214,168x + 27,102, *R*^2^ = 0. 9999; LD = 0.14 μg/mL; LQ = 0.52 μg/mL, peak 1); apigenin-6-C-glucoside (y = 197,337x + 30,036, LD = 0. 19 μg/mL; LQ = 0.63 μg/mL, peaks 8, 9, 10, and 12); (-)-catechin (y = 84.950x − 23.200, *R*^2^ = 0.999, LD = 0.17 μg/mL; LQ = 0.68 μg/mL, peaks 11, 14, 15, 17, 18, 20 and 21); naringenin (y = 18,433x + 78,903, LD = 0. 17 μg/mL; LQ = 0.81 μg/mL, peaks 13, 16, and 19); and quercetin-3-O-glucoside (y = 34,843x − 160,173, LD = 0.21 μg/mL; LQ = 0.71 μg/mL, peaks 6 and 7). All peaks showed a *p*-value < 0.001 for the Student’s *t*-test.

## Data Availability

Data are contained within the article.

## References

[1] Stutts L.R., Vermerris W. (2020). Elucidating Anthracnose Resistance Mechanisms in Sorghum—A Review. Phytopathology.

[2] Tolentino D.C., Rodrigues J.A.S., Pires D.A.d.A., Veriato F.T., Lima L.O.B., Moura M.M.A. (2016). The quality of silage of different sorghum genotypes. Acta Sci..

[3] Weerasooriya D.K., Bean S.R., Nugusu Y., Ioerger B.P., Tesso T.T. (2018). The effect of genotype and traditional food processing methods on in-vitro protein digestibility and micronutrient profile of sorghum cooked products. PLoS ONE.

[4] Marchini M., Marti A., Folli C., Prandi B., Ganino T., Conte P., Fadda C., Mattarozzi M., Carini E. (2021). Sprouting of Sorghum (*Sorghum bicolor* [L.] Moench): Effect of Drying Treatment on Protein and Starch Features. Foods.

[5] Ratnavathi C.V. (2019). Grain Structure, Quality, and Nutrition. Breeding Sorghum for Diverse End Uses.

[6] Crozier D., Riera-Lizarazu O., Rooney W.L. (2022). Application of X-ray computed tomography to analyze the structure of sorghum grain. Plant Methods.

[7] Queiroz V.A.V., Silva C.S., Menezes C.B., Schaffert R.E., Guimaraes F.F.M., Guimaraes L.J.M., Guimaraes P.E.O., Tardin F.D. (2015). Nutritional composition of sorghum [*Sorghum bicolor* (L.) Moench] genotypes cultivated without and with water stress. J. Cereal Sci..

[8] Espitia-Hernández P., Chávez González M.L., Ascacio-Valdés J.A., Dávila-Medina D., Flores-Naveda A., Silva T., Ruelas Chacón X., Sepúlveda L. (2022). Sorghum (*Sorghum bicolor* L.) as a potential source of bioactive substances and their biological properties. Crit. Rev. Food Sci. Nutr..

[9] Kang J., Price W.E., Ashton J., Tapsell L.C., Johnson S. (2016). Identification and characterization of phenolic compounds in hydromethanolic extracts of sorghum wholegrains by LC-ESI-MSn. Food Chem..

[10] Palacios C.E., Nagai A., Torres P., Rodrigues J.A., Salatino A. (2021). Contents of tannins of cultivars of sorghum cultivated in Brazil, as determined by four quantification methods. Food Chem..

[11] Wu Y., Li X., Xiang W., Zhu C., Lin Z., Wu Y., Li J., Pandravada S., Ridder D.D., Bai G. (2012). Presence of tannins in sorghum grains is conditioned by different natural alleles of *Tannin1*. Proc. Natl. Acad. Sci. USA.

[12] Xu J., Wang W., Zhao Y. (2021). Phenolic Compounds in Whole Grain Sorghum and Their Health Benefits. Foods.

[13] Saithalavi K.M., Bhasin A., Yaqoob M. (2021). Impact of sprouting on physicochemical and nutritional properties of sorghum: A review. J. Food Meas. Charact..

[14] Xu M., Jin Z., Simsek S., Hall C., Rao J., Chen B. (2019). Effect of germination on the chemical composition, thermal, pasting, and moisture sorption properties of flours from chickpea, lentil, and yellow pea. Food Chem..

[15] Ocheme B.O., Adedeji E.O., Lawal G., Zakari M.U. (2015). Effect of Germination on Functional Properties and Degree of Starch Gelatinization of Sorghum Flour. J. Food Res..

[16] Liu S., Wang W., Lu H., Shu Q., Zhang Y., Chen Q. (2022). New perspectives on physiological, biochemical and bioactive components during germination of edible seeds: A review. Trends Food Sci. Technol..

[17] Singh A., Sharma S., Singh B. (2017). Effect of germination time and temperature on the functionality and protein solubility of sorghum flour. J. Cereal Sci..

[18] Leite D.D.F., Cavalcanti M.T., Silva A.S., Gonçalves M.C., Almeida M.C.B.M. (2016). Propriedades funcionais da semente do sorgo (*Sorghum bicolor* (L.) Moench) in natura e germinado. Rev. Verde Agroecol. Desenvolv. Sustent..

[19] Contreras-Jiménez B., Del Real A., Millan-Malo B.M., Gaytán-Martínez M., Morales-Sánchez E., Rodríguez-García M.E. (2019). Physicochemical changes in barley starch during malting. J. Inst. Brew..

[20] AACC (2000). Approved Methods of the American Association of Cereal Chemists.

[21] Guerra-Oliveira P., Belorio M., Gómez M. (2022). Wasted bread flour as a novel ingredient in cake making. Int. J. Food Sci. Technol..

[22] Beuchat L.R. (1977). Functional and electrophoretic characteristics of succinylated peanut flour protein. J. Agric. Food Chem..

[23] Köhn C.R., Fontoura A.M., Kempka A., Demiate I., Kubota E.H., Prestes Dornelles R. (2015). Assessment of different methods for determining the capacity of water absorption of ingredients and additives used in the meat industry. Int. Food Res. J..

[24] Lin M.J.Y., Humbert E.S., Sosulski F.W. (1974). Certain Functional Properties of Sunflower Meal Products. J. Food Sci..

[25] Spier F., Zavareze E.d.R., Marques e Silva R., Elias M.C., Dias A.R.G. (2013). Effect of alkali and oxidative treatments on the physicochemical, pasting, thermal and morphological properties of corn starch. J. Sci. Food Agric..

[26] Curti M.I., Belorio M., Palavecino P.M., Camiña J.M., Ribotta P.D., Gómez M. (2022). Effect of sorghum flour properties on gluten-free sponge cake. J. Food Sci. Technol..

[27] AOAC (1999). Official Methods of Analysis of AOAC International.

[28] UNIÃO EUROPEIA (2011). Regulamento (UE) No. 1169/2011 do Parlamento Europeu e do Conselho de 25 de Outubro de 2011 Relativo à Prestação de Informação aos Consumidores Sobre os Qéneros Alimentícios. https://www.proquest.com/openview/9e7994d22db38bb29f9a5ebc50704420/1?pq-origsite=gscholar&cbl=2026366&diss=y.

[29] Barros L., Pereira E., Calhelha R.C., Duẽnas M., Carvalho A.M., Santos-Buelga C., Ferreira I.C.F.R. (2013). Bioactivity and chemical characterization in hydrophilic and lipophilic compounds of Chenopodium ambrosioides L. J. Funct. Foods.

[30] Souilem F., Fernandes Â., Calhelha R.C., Barreira J.C.M., Barros L., Skhiri F., Martins A., Ferreira I.C.F.R. (2017). Wild mushrooms and their mycelia as sources of bioactive compounds: Antioxidant, anti-inflammatory and cytotoxic properties. Food Chem..

[31] Lockowandt L., Pinela J., Roriz C.L., Pereira C., Abreu R.M.V., Calhelha R.C., Alves M.J., Barros L., Bredol M., Ferreira I.C.F.R. (2019). Chemical features and bioactivities of cornflower (*Centaurea cyanus* L.) capitula: The blue flowers and the unexplored non-edible part. Ind. Crops Prod..

[32] Mandim F., Petropoulos S.A., Dias M.I., Pinela J., Kostic M., Soković M., Santos-Buelga C., Ferreira I.C.F.R., Barros L. (2021). Seasonal variation in bioactive properties and phenolic composition of cardoon (*Cynara cardunculus* var. altilis) bracts. Food Chem..

[33] Bessada S.M.F., Barreira J.C.M., Barros L., Ferreira I.C.F.R., Oliveira M.B.P.P. (2016). Phenolic profile and antioxidant activity of *Coleostephus myconis* (L.) Rchb.f.: An underexploited and highly disseminated species. Ind. Crops Prod..

[34] Sajdakowska M., Gębski J., Żakowska-Biemans S., Jeżewska-Zychowicz M. (2019). Willingness to eat bread with health benefits: Habits, taste and health in bread choice. Public. Health.

[35] Dong Y., Karboune S. (2021). A review of bread qualities and current strategies for bread bioprotection: Flavor, sensory, rheological, and textural attributes. Compr. Rev. Food Sci. Food Saf..

[36] Garvey E.C., O’Sullivan M.G., Kerry J.P., Kilcawley K.N. (2020). Factors influencing the sensory perception of reformulated baked confectionary products. Crit. Rev. Food Sci. Nutr..

[37] Guiné R.P.F. (2022). Textural Properties of Bakery Products: A Review of Instrumental and Sensory Evaluation Studies. Appl. Sci..

[38] Liu T., Hou G.G., Cardin M., Marquart L., Dubat A. (2017). Quality attributes of whole-wheat flour tortillas with sprouted whole-wheat flour substitution. LWT.

[39] Olamiti G., Takalani T.K., Beswa D., Jideani A.I.O. (2020). Effect of malting and fermentation on colour, thermal properties, functional groups and crystallinity level of flours from pearl millet (*Pennisetum glaucum*) and sorghum (*Sorghum bicolor*). Heliyon.

[40] Martino H., Tomaz P., Moraes É., Conceição L., Oliveira D., Queiroz V., Rodrigues J., Pirozi M., Pinheiro-Sant'Ana H., Ribeiro S. (2012). Chemical characterization and size distribution of sorghum genotypes for human consumption. Rev. Inst. Adolfo Lutz.

[41] Jribi S., Sahagùn M., Debbabi H., Gomez M. (2019). Evolution of functional, thermal and pasting properties of sprouted whole durum wheat flour with sprouting time. Int. J. Food Sci. Technol..

[42] Rothschild J., Rosentrater K., Onwulata C., Singh M., Menutti L., Jambazian P., Omary M. (2015). Influence of quinoa roasting on sensory and physicochemical properties of allergen-free, gluten-free cakes. Int. J. Food Sci. Technol..

[43] Lin S., Gao J., Jin X., Wang Y., Dong Z., Ying J., Zhou W. (2020). Whole-wheat flour particle size influences dough properties, bread structure and in vitro starch digestibility. Food Funct..

[44] Ma Z., Marsolais F., Bernards M.A., Sumarah M.W., Bykova N.V., Igamberdiev A.U. (2016). Glyoxylate cycle and metabolism of organic acids in the scutellum of barley seeds during germination. Plant Sci..

[45] Li C., Oh S.-G., Lee D.-H., Baik H.-W., Chung H.-J. (2017). Effect of germination on the structures and physicochemical properties of starches from brown rice, oat, sorghum, and millet. Int. J. Biol. Macromol..

[46] He Y., Song S., Li C., Zhang X., Liu H. (2022). Effect of germination on the main chemical compounds and 5-methyltetrahydrofolate metabolism of different quinoa varieties. Food Res. Int..

[47] Zhang G., Xu Z., Gao Y., Huang X., Zou Y., Yang T. (2015). Effects of Germination on the Nutritional Properties, Phenolic Profiles, and Antioxidant Activities of Buckwheat. J. Food Sci..

[48] Igamberdiev A.U., Eprintsev A.T. (2016). Organic Acids: The Pools of Fixed Carbon Involved in Redox Regulation and Energy Balance in Higher Plants. Front. Plant Sci..

[49] Beltrão N.E.d.M., de Oliveira M.I.P. (2007). Biossíntese e Degradação de Lipídios, Carboidratos e Proteínas em Oleaginosas. Embrapa Algodão-Documentos (INFOTECA-E). https://www.infoteca.cnptia.embrapa.br/bitstream/doc/275924/1/DOC178.pdf.

[50] Zhang Q., Peng S., Li Y., Zhang H., Qin X., Liu G. (2023). Malic acid enhances proanthocyanidin stability and their combined effects on dough rheological properties and bread quality. LWT.

[51] Su X., Wu F., Zhang Y., Yang N., Chen F., Jin Z., Xu X. (2019). Effect of organic acids on bread quality improvement. Food Chem..

[52] Li X., Li J., Dong S., Li Y., Wei L., Zhao C., Li J., Liu X., Wang Y. (2019). Effects of germination on tocopherol, secoisolarlciresinol diglucoside, cyanogenic glycosides and antioxidant activities in flaxseed (*Linum usitatissimum* L.). Int. J. Food Sci. Technol..

[53] Sruthi N.U., Rao P.S. (2021). Effect of processing on storage stability of millet flour: A review. Trends Food Sci. Technol..

[54] Barden L., Decker E.A. (2016). Lipid Oxidation in Low-moisture Food: A Review. Crit. Rev. Food Sci. Nutr..

[55] Rios R.V., Pessanha M.D.F., de Almeida P.F., Viana C.L., Lannes S.C. (2014). da S. Application of fats in some food products. Food Sci. Technol..

[56] Pinheiro S.S., Anunciação P.C., de Morais-Cardoso L., Della Lucia C.M., de Carvalho C.W.P., Queiroz V.A.V., Sant'Ana H.M.P. (2021). Stability of B vitamins, vitamin E, xanthophylls and flavonoids during germination and maceration of sorghum (*Sorghum bicolor* L.). Food Chem..

[57] Irondi E.A., Adegoke B.M., Effion E.S., Oyewo S.O., Alamu E.O., Boligon A.A. (2019). Enzymes inhibitory property, antioxidant activity and phenolics profile of raw and roasted red sorghum grains in vitro. Food Sci. Hum. Wellness.

[58] Pontieri P., Pepe G., Campiglia P., Merciai F., Basilicata M.G., Smolensky D., Calcagnile M., Troisi J., Romano R., Giudice F. (2021). Comparison of Content in Phenolic Compounds and Antioxidant Capacity in Grains of White, Red, and Black Sorghum Varieties Grown in the Mediterranean Area. ACS Food Sci. Technol..

[59] Wu G., Johnson S.K., Bornman J.S., Bennett S.J., Fang Z. (2017). Changes in whole grain polyphenols and antioxidant activity of six sorghum genotypes under different irrigation treatments. Food Chem..

[60] Wu G., Bennett S.J., Bornman J.F., Clarke M.W., Fang Z., Johnson S.K. (2017). Phenolic profile and content of sorghum grains under different irrigation managements. Food Res. Int..

[61] Xiong Y., Zhang P., Warner R.D., Fang Z. (2019). Sorghum Grain: From Genotype, Nutrition, and Phenolic Profile to Its Health Benefits and Food Applications. Compr. Rev. Food Sci. Food Saf..

[62] Xiong Y., Damasceno Teixeira T.V., Zhang P., Warner R.D., Shen S., Fang Z. (2021). Cellular antioxidant activities of phenolic extracts from five sorghum grain genotypes. Food Biosci..

[63] Cáceres P.J., Martínez-Villaluenga C., Amigo L., Frias J. (2014). Maximising the phytochemical content and antioxidant activity of Ecuadorian brown rice sprouts through optimal germination conditions. Food Chem..

